# From Lay Depression Narratives to Secular Ritual Healing: An Online Ethnography of Mental Health Forums

**DOI:** 10.1007/s11013-020-09702-5

**Published:** 2020-12-28

**Authors:** Domonkos Sik

**Affiliations:** Department of Social Theory, University of Eötvös Loránd, Pázmány Péter sétány 1/a, Budapest, 1117 Hungary

**Keywords:** Depression, Online forums, Biopower, Ritual healing, Online ethnography

## Abstract

The article aims at analysing online depression forums enabling lay reinterpretation and criticism of expert biomedical discourses. Firstly, two contrasting interpretations of depression are reconstructed: expert psy-discourses are confronted with the phenomenological descriptions of lay experiences, with a special emphasis on online forums as empirical platforms hosting such debates. After clarifying the general theoretical stakes concerning contested ‘depression narratives’, the results of an online ethnography are introduced: the main topics appearing in online discussions are summarised (analysing how the abstract tensions between lay and expert discourses appear in the actual discussions), along with the idealtypical discursive logics (analysing pragmatic advises, attempts of reframing self-narratives and expressions of unconditional recognition). Finally, based on these analyses an attempt is made to explore the latent functionality of online depression forums by referring to a secular ‘ritual healing’ existing as an unreflected, contingent potential.

## Introduction

Depression is a concept elaborated in the biomedical and psy-discourses. This embeddedness has a special significance, as the depressive phenomenological patterns (Ratcliff 2015) are inseparable from the objectifying and alienating tendencies of modernisation (Rosa 2010). From the perspective of the critique of biopower (Foucault 1997; Rose 1999), it may be argued that the reifying interpretations and praxes grounding modern biomedical institutions are tainted by the same distortions and paradoxes, which are responsible for depression as social suffering in the first place (Sik 2019). Such ambiguities express the narrative challenges of overcoming depression: the affected actors have to deal not only with depressed mood, but also with the objectifying biomedical interpretative framework—a task requiring appropriate discursive spaces.

The present article aims to explore these narrative struggles by analysing the manifest and latent communicative processes of online depression forums with the tools of online ethnography. These platforms are understood as complex public spheres mediating between expert and lay positions: while their manifest function is information exchange and peer support, they fulfil latent functions as well. These considerations outline the structure of the following analysis. Firstly, the biomedical interpretation of depressed mood (expressing an objectifying expert perspective) is confronted with a phenomenological one (expressing a lay narrative universe), with a special focus on online forums as platforms hosting such debates. Secondly, an online ethnography is elaborated: the main narratives and discursive strategies of depression forums are overviewed; also a latent functionality of ‘ritual healing’ (Csordas 1983) is described in a short case study.

## Theoretical Grounding: Seeking Non-reifying Depression Narratives in the Online Sphere

Despite its positivist self-understanding, biomedicine is inseparable from the discursive structures of biopower (Foucault 1997). Since biomedical discourse became a dominant language of suffering, its authority was less and less limited to clinical context. Gradually normalising technologies of the self were elaborated focusing on the right conduct of everyday life from the perspective of healthiness (Rose 1999: 74). These biomedical frameworks did not simply complement the already existing cultural or moral ones, but—by claiming to express the ‘objective truth’ of human nature—marginalised and silenced the alternatives. Consequently, only those aspects of suffering were recognised, which proved to be translatable to the biological language games (Kleinman and Kleinman 1991). This exclusivity is required for grounding an undeniable aura of authority: biomedical discourse implies that most forms of suffering are translatable to medical terms and also solvable with medical technologies (Good 2007). The biomedical reduction of suffering not only resulted in the elimination of personal narratives responsible for making sense of the suffering, but also in alienated self-understanding making the subject a helpless spectator of their own misery (Kleinman 1973). As such reductionist approach proves to be inefficient in case of various forms of suffering, it is complemented by technologies of actual objectification. As the overuse of painkillers or anti-depressants proves it, if every attempts of treating the cause of suffering fail, the final solution of biomedical paradigm is the sedating of the sufferer that is depriving them from the chance of reflecting upon their condition (Hardon and Sanabria 2017).

The silencing of sufferers turned into passive patients did not however eliminate the need for personal interpretation. This is reflected in the various criticisms concerning the biomedical framing of mental suffering (e.g. Scheff 1999; Laing-Esterson 1964). Being criticised from outside and inside, biomedical psychiatry has lost its aura of unquestionable authority probably more than other medical fields (Rose and Abi-Rached 2013; Rose 2018). In case of mental disorders, the biomedical discourse is not only more contested by alternative ones, but also the importance of lay interpretations is greater. These conclusions highlight the stakes of online depression forums, which are distinguished platforms of such interpretative struggles.

### Depression: Biomedical Versus Phenomenological Model

The notion of depression is elaborated in a wide clinical discourse including disciplines such as neurology, psychiatry or the various schools of psychology. Even if these approaches represent a diverse ontological, epistemic and therapeutic universe, they share the basic assumptions of biomedical paradigm by interpreting depression as an objectively describable—physical or mental—pathology (APA 2013: 160–164; Lewinsohn et al. 1979; Beck 1967; Seligman 1975). From such approach comes an objectifying reductionism, as the expert interpretation of depression tends to ignore the phenomenological complexity of the actual experience. By applying reductionist descriptions of suffering, it not only risks the failure of finding adequate treatments, but also the creation of problems originating from the alienating interpretation itself. This explains the birth of phenomenological approaches focusing on the first person accounts of the sufferers.

Depression affects the totality of ‘being in the world’: an inhabitable environment is turned into an uninhabitable world, full of obstacles (van der Berg 1972: 45). The experience of depression is characterised by the distortion of the horizon of both the future (i.e. hopelessness) and the past (i.e. guilt). As a consequence of being dropped out from time, meaningful action gives its place to passivity and ignorance (Ratcliff 2015: 170). The withdrawal from activity results in the disconnecting from the shared material world (intercorporality), intersubjective time (intertemporality) and collective emotions (interaffectivity). These experiences do not simply imply solitude, but also the inability of relating to the other that is being deprived from intersubjectivity. If intersubjectivity is suspended on the elementary level, then the subject is stuck in an ambiguous condition, ‘neither alive nor dead’ (Fuchs 2013: 229).^1^ These experiences can be compared to the biomedical descriptions of depression in order to understand the lay actors’ perplexity when applying expert narratives.

On the one hand, representatives of biomedical discourse frame depression as an inherent–either bodily or mental–pathology of the actor: an objective condition being treatable according to expert knowledge concerning the pathology. On the other hand, lay sufferers experience a burdensome world, a frozen time, an immobilised self and unrelatable others. From these interpretations, two contrasting trajectories of treatment follow: while biomedical paradigm is based on bodily intervention or the correction of cognitive, emotive and behavioural habits, phenomenology emphasises the need for reintegration into an intersubjective being in the world that is the revitalisation of the sufferer through intercorporeal interactions re-establishing interaffectivity (Fuchs-Koch 2014). Such discrepancy has central relevance: in therapeutic interactions the objectifying gaze of the experts becomes a further reinforcement of a world, where the depressed subject could exist only as a helpless object. The very act of biomedical therapy contributes to the feeling of detachment, instead of being able to reconstruct intersubjectivity. In this sense, independently from the effectivity of the medical or therapeutic treatment, the biomedical framing of the sufferer reinforces a phenomenological horizon similar to the ones being responsible for the emergence of depression in the first place.

Consequently, biomedical framing not only reduces the depressive experience into a pattern of biological and mental symptoms, but more importantly creates an inacceptable narrative for the sufferer, which further reinforces the sense of remoteness. Accordingly, biomedical knowledge inevitably plays an ambiguous role in the process of dealing with depressive experiences, which is expressed in the tension between lay and expert discourses: while providing therapies, because of its reifying gaze, biomedicine further blocks the emergence of an undistorted intersubjectivity. In this constellation a quest for ‘illness narratives’ (Kleinmann 1988; Frank 2013) begins. Online depression forums play an important role in these processes: they are micro-level platforms for the troubled subjectivities seeking answers to personal existential questions.

### The Narrative Universe of Online Depression Forums

The lay narratives of depression have been analysed in various contexts. The experience of any unexpected, uncontrollable illness creates fissure on the actor’s identity. The primary function of lay illness narratives is to restore a coherent self by providing a meaningful explanation for a being in the world burdened by illness (Kleinmann 1988: 48). In case of depression, the particular challenge of finding such narratives is related to the ambiguous status of the distorted mood itself: from a first person perspective, mood is considered to be the essential part of the self, which implies moral responsibility (i.e. bad mood is to be corrected by the actors themselves); from the expert perspective, it is considered to be a pathology implying external intervention. In this sense, depression challenges the self on an elementary level: the actors have to choose between not identifying with the distorted mood (thus escape moral responsibility at the cost of losing a constitutive element of the self) or identifying with it (thus keeping the integrity of the self at the cost of a negative self-evaluation). There is no win–win solution to such dilemma, which means that either option implies the giving up on important elements of the self. Thus, the narrative challenge of depression is to minimise such losses (Flynn 2010). According to several interview-based researches, the typical narrative strategies rely on idealtypical dramaturgical steps: the pre-depression past is introduced as golden age compared to the present; a dramatic event is introduced as a starting point of troubles; the inhabiting of a narrow space of agency is described; if there is successful dealing with depression a phase of confession to others is mentioned, along with the lessons learned (Hajela 2012). It may be argued that these narratives highlight the stakes of the clash between lay and expert discourses that is the expression of the actors’ attempt to poise between agency and responsibility.

While these narratives were gathered in offline context, they have relevance for online discourses as well. Firstly, however it has to be clarified in what extent is online communication capable of enabling the construction complex narratives. Since the antiquity, the public sphere has been a terrain of equals, who form their cooperation without any pre-given hierarchy, purely based on the convincing and affecting of each other (Arendt 1998). The recent rise of information society however has transformed the public sphere as well: while it provided access to previously unseen masses, it also reduced the quality of communication to a level, where rational discussion is barely possible (Davis 2020). Being part of such ‘anti-public sphere’, depression forums have to face not only the challenges of biomedical hegemony, but also the ones originating from the difficulties of online communication. According to the criticism of information society, the very logic of information is in contradiction with narratives. The latter requires time, which is limited by the continuously streamed ephemeral bits of information (Lash 2002).

While such diagnoses certainly highlight a limitation of online communication, several empirical studies show that online communication is capable of reproducing important elements of face-to-face interactions. Given enough time, the partners may develop codes capable of transmitting emotions and latent meanings similar to non-verbal communication. Of course, not every online platform succeeds in maintaining a confidential, lively discussion. The key factors of success include on the one hand the safeguard of the quality of public sphere: moderation in order to filter offensive and toxic posts; a subtle balancing between anonymity and identification (in order to avoid both recognisability and unreliability at the same time); and the maintaining of boundaries. On the other hand, success depends on the expected positive outcomes, such as the chance of individual catharsis; the support of personal coping strategies; and the experience of being embedded in a supportive community (Murphy et al. 2020).

Even if mediated communication is less intense and its impact is less durable, these contingencies may become advantages in case of sensitive topics, which are harder to be discussed directly. In case of sharing negative feelings, anonymity may be reassuring, and thus online platforms may be considered as secure spaces (Derks et al. 2008). Besides the potential of communicating emotions, which is the prerequisite of re-establishing interaffectivity, online communication is also capable of providing community experience (Galegher 1998). Furthermore, participating in online communication has therapeutic potential by providing a safe, low-risk space of self-disclosure (Benzon 2008). Online public spheres focusing on depression rely on all of these advantages by providing a safe space for experimenting with various lay narratives. These platforms function as ‘narrative sandboxes’, where an attempt is made to express the experience of depression, to re-enter intersubjectivity in a low threshold interaction and find mutual explanations to the suffering (Kotliar 2015). In case of online forums, not only the experimenting with various narratives is available in front of a virtual audience, but also support through communication (Prescott et al. 2017): as various participants define their own role differently, such support could equally manifest as a direct attempt (that is telling the other what to do) or an indirect one (i.e. giving advice by telling a similar personal story, while expressing sympathy and understanding). Overall, it seems that despite its limitations, online communication has a potential of elaborating identity-narratives.

## The Quest for Meaning: Manifest and Latent Functionalities of Online Depression Forums

Online mental health forums represent an expanding sphere. Previous researches have shown that the main motivation for using them is threefold: it helps to overcome social and geographical isolation; it enables establishing social relationships, which are missing from the participants’ life; and it provides opportunity for gathering practical information and advice (Smith-Merry et al. 2019). While the majority of the users suffer from depressed mood (Powell et al. 2003), the participants of online depression forum should be viewed as a heterogeneous population. According to an online survey, at least four idealtypical groups can be differentiated (Nimrod 2013): ‘concerned about daily living’ (mostly female users, higher depression scale scores), ‘information seekers’ (mostly men, infrequent users), ‘interested in all topics’ (mostly female frequent users) and ‘relatively less involved’ (mostly male, frequent users). Due to these variabilities, it should be noted that the population of the forums does not cover or represent the clinical population of those who suffer from depression; also the narratives explored cover only the self-expression of those who choose to express themselves, not the community as a whole. Keeping these limitations in mind, an online ethnography was conducted to reveal those less obvious, latent mechanisms and functionalities, which organise the forum discussions.

### Methods, Ethical Concerns and Data

As online ethnography is a relatively new subfield, its methodological and ethical grounds are yet to become consolidated. Ethnography in general aims at analysing the social and cultural patterns through observing—and often participating in—social praxes in a systematic way, that is to provide a description of the actors’ meanings and way of life, along with the interactions reproducing them. Exploring a lifeworld from the inside implies the ethnographer’s involvement in the social interactions themselves. Such challenging task requires epistemological reflection on the role of the researcher in a given community (Wolcott 1999). As online ethnography is conducted in a virtual space, these general features also need to be accommodated to the specific environment. Probably the most important difference concerns the ‘façade’ of the actors (Goffman 1959): they are not present in their physical reality, only as ‘virtual avatars’ existing within the technological framework of the online platforms. While technology is not irrelevant in offline encounters either, it does not necessarily play a decisive role, unlike in case of online interactions, where the subjects appear only as mediatised personas. Accordingly, online ethnography inevitably deals with ‘actor-networks’ (Latour 2005) constituted of human and non-human elements playing an equally constitutive role. While the analysis of mediated interactions is characterised by obvious limitations (e.g. the inaccessibility of the offline social and cultural context of the avatars), it also provides unique opportunities, such as the extra-temporal position of the observer. As most interactions are archived, the antecedents and the reactions to any speech acts are accessible, which creates opportunity for reconstructing idealtypical dynamics in their temporal totality.

Online ethnography also raises ethical questions of its own. Although there are several ethical guidelines about doing such research (e.g. BPS 2017; AoIR et al. 2012), as each field has unique features, decisions fitting the specific context have to be made. Besides these general guidelines, the examples of previous similar research may also help an informed decision. As a general rule, it may be claimed that ‘The greater the vulnerability of the community, the greater the obligation of the researcher to protect it’ (AoIR et al. 2012: 4). Being a mental disorder, depression is still surrounded by many misunderstandings, controversies and stigma, which further increase the burdens of those who are affected by it. Accordingly, even if participants of online depression forums cannot be viewed as a clinical population, sill they have to be treated as belonging to a potentially vulnerable group. This feature affected our ethical and methodological decisions in many ways.

Firstly, it was decided that solely those materials are to be accessed, which were anonymously posted, do not require subscription, login or password. As these posts are intended to be publicly viewed (a consent is given by accepting the ‘terms of use’), there is wide ethical consensus that their access does not require a specific approval from the participants, the administrators of the forum, or a specific approval from an IRB (Kotliar 2015: 4; O’Brien and Clark 2012: 279; Salzmann-Erikson and Eriksson 2012: 11). However, despite this wide consensus, the status of sensitive public forums still raises questions. Every time private matters are discussed in public forums, the dividing line between the two spheres blurs, which implies the need for specific caution (BPS 2017: 7). Depending on the field, several decisions are to be made (Salzmann-Erikson and Eriksson 2012: 12): an overt (advertising the fact of observation) or covert (nod disclaiming the process of observation) field work may be chosen; in the process of publication, the participants or even the fields may be highlighted (e.g. by providing links) or concealed (by using anonyms of the avatars or modifying the citations).

While making these decisions, the principles of ‘Respect for the autonomy, privacy and dignity of individuals and communities; Scientific integrity; Social responsibility; Maximising benefits and minimising harm’ equally have to be taken into consideration (BPS 2017: 4). In our case, the vulnerability of the researched group leads to a maximally secure strategy: in accordance with these principles a covert, non-participant observation was applied, while the results are being presented in a completely concealed way. As the forums are visited by many people in severe distress seeking advice or help, any research should respect their protection in the first place. As the consequences of any form of participation are unpredictable in a virtual space, where the background and the reactions of the persons behind the avatars are inaccessible, attempts of intervention were avoided in order to eliminate the risk of unintentional harms. Furthermore, advertising the fact of the observation has the potential of reconfiguring the locally established frames of supportive interactions, thus not only discouraging participation, but also distorting the interactions themselves. In our case, the ‘contaminating of the field’ risks not only the loss of original knowledge otherwise inaccessible (Salzmann-Erikson and Eriksson 2012: 11), but even more so, the endangering of the supportive interactions enabled by depression forums. In order to prevent these dangers, the non-participant observation was executed covertly. Of course, such decision is justifiable only, if the subjects of observation remain completely unidentifiable: this means not only the elimination of the traces of avatars (by changing the nicknames and the citations), but also the anonymisation of the forums themselves.^2^ This way the dangers related to the widespread dissemination of the results (BPS 2017: 17) can also be minimised (as neither the communities nor the avatars are identifiable).

Based on these premises, an online ethnography of several English-speaking mental health forums was conducted, which partly or completely focus on depression.^3^ Data were systematically gathered and analysed with the method of ‘deep reading’ (Lee 2017; Skageby 2010). In case of each forum, the new threads were followed for a month in three consecutive waves (in 2018 November, 2019 Mach and 2019 September). The field work was finished after the third wave, because of saturation (as new narratives and discursive logics did not show up any longer). During these timeframes, posts were categorised according to the substantive topic (e.g. problems with medication, therapy, etc.) and the discursive role they play (e.g. descriptive, argumentative, etc.). Also, the dynamics of the relevant threads were analysed according to the manifest (e.g. sharing one’s personal story) and latent (e.g. supporting the other) components. Based on these initial analyses, various aspects of the everyday online praxes were elaborated: the substantive topics as they are revealed for a newcomer; the types of dialogues as they are experienced by adept users forming community ties; the hidden mechanisms, which constitute quasi-ritual patterns.

### Entering and Exploring: Thematic Clusters of Depression Forums

Most visitors of the forums are affected by depression either directly or indirectly. Accordingly, my role as an observant also had to be clarified. The whole rhythm and praxis of the ethnography depends on this: depression forums are used differently by the newcomers having practical questions; shy users wandering around without any concrete purpose; experienced members being involved in one or another type of discursive praxes on a regular basis; and the ‘elders’ attempting to provide support for those in need. At the beginning, I identified as a shy newcomer, who is indirectly affected by depression: I wanted to know as much about it, as possible with the help of the forum community, without actively taking part in the conversations. I was particularly interested in the reception and the collective reinterpretation of expert discourses. I started to visit the forums as an explorer trying to map the available threads. The technological infrastructure of the forums seemed to shorten this process, as discussions are organised in a complex classificatory system. Although these categories definitively help navigation in the first few times, they become less relevant as one gets familiar with the environment. As one’s interest becomes clearer, the key topics and the relevant others become the main reference points. Based on my first experiences, a ‘thematic map’ was prepared (see Appendix), indicating the recurring topics and the idealtypical forms of narration.

The overview of the thematic clusters and idealtypical narratives highlighted how the expert discourses of depression clash with the lay experiences. Biomedical explanations appear in a fundamentally ambiguous framework. Among the causes of depression bodily causes are embedded in a broader context of lifestyle, framing the body as an intermediator of external effects instead of being a final *explanans*. Biomedical interventions, while generally approved, are also considered to have limited efficiency: the experts’ authority is questioned for several reasons; medications are considered to be highly contingent and potentially dangerous, inefficient on their own; they are rather experimented during the therapeutic process, instead of being applied according to verified protocols. The sharing of experiences of ‘negative automatic thoughts’ constitutes a significant part of the discussions: attempts are made to distance them from the self; their logic is deconstructed in order to reveal the hidden paralysing loops. Instead of relying on processes inaccessible to the actors, most narratives of distorted development refer to an environment being responsible for restricting agency. Accordingly, even if psychological explanations are evoked, they are complemented with a social perspective: strategies of control through self-harm, escapism or disconnecting are explained according to an ignorant, intolerant or discriminating social context. Traumas appear as examples of dramatic events turning the subject’s being in the world inside out. Unlike in expert discourses however, they do not paralyse the actors, instead provide opportunity to name those, who are responsible for the suffering.

Besides complementing biological and psychological explanations with narratives of a dysfunctional social environment, social distortions also appear on their own right. Burdensome social norms and expectations reveal those symbolic obligations, which set impossible tasks for the individual. Personal failures of succeeding in economic competition or in the struggle for recognition indicate how the experience of losing in the ruthless everyday battles contributes to the emergence of depression. As one’s existence is both completely dependent on social worthiness, being proved to be unworthy in various material and symbolic fields threatens with the total dissolving of identity. Finally, the distortions of communities and society are also indicated as a source of suffering: if family and intimate relationships are dysfunctional, they provide space only for burdensome interactions; if the work relations are insecure or exploitative, hopelessness becomes a general experience; if collective action seems to fail on a fundamental level, personal suffering appears as a consequence of universal paradoxes.^4^

Overall, it may be argued that online depression forums host discussions, which are partly relativising biomedical explanations; partly complementing psy-diagnoses with social ones; partly link the experience of suffering to various dysfunctions or distortions of the social world directly. Accordingly, these forums serve as discursive spaces capable of complementing expert interpretations with lay ones. However, besides such interpretative function they also enable other mechanisms. In order to reveal them, the dynamics of interactions has to be analysed.

### Approaching Online Communities: The Discursive Dynamics

As one is getting familiar with the online platform and the topics, the approach to the forums also transforms. It relies less on the general classificatory categories provided by the interface; instead customised shortcuts are established, which directly lead to the relevant discussions and the significant others. By spending some time with the online community, it becomes clear that most threads have a short life expectancy: in case of targeted questions, concrete answers end the discussion; in case of insecure participants, the first few generic welcoming posts are often not enough to motivate a deeper disclosure. So the more experienced users usually start to move together: they follow each other through various topics and create a subtext of their own within threads dealing with various issues. At this point, the contours of online communities become visible. After getting familiar with the forum, the second step of initiation involves following one of these quasi-groups. From this perspective, a new dimension of the forum life becomes accessible: beyond the substantive topics, idealtypical discursive strategies appear, which integrate loose networks of experienced users forming virtual communities.

In the process of group formation, institutionalised tokens indicating one’s status play a central role. Several forums enable not only the customisation of avatars, but also their decoration with visual indicators expressing one’s rank (which is often based on quantitative factors, such as the length of membership, the number of posts, or ‘likes’ given by other members). This way a differentiation—and a struggle for recognition—among the participants may occur resulting in the emergence of specific roles and statuses. Such roles include the basic differentiation between the ‘newcomer’ looking for advice, the ‘experienced members’ and the ‘respected elders’, who often act as ‘recovering helpers’ (Rácz et al. 2015). These various roles enable different discursive strategies: newcomers usually remain in the position of seeking advice; the experienced members may help in the process of narrative reinterpretation; the elders combine personal experience and expertise, which is not based on formal training, but the successful dealing with the suffering.

The first idealtypical role is exemplified by the following discussion:

Newcomer1: Hi, I have recently been prescribed a low dose of antidepressants, these are to try and help me with my low mood. This is the 2nd day of taking them and both days I have been really tired and grouchy in the evening, is this being caused by the tablets do you guys think? I just feel really lazy and that I’m going to be stuck like this forever now :))

Experienced member1: What time of day do you take the tablets?

Newcomer1: In the morning usually about 10am

Experienced member1: In that case it’s very likely that the medication is wearing off by the evening, particularly if you’re on a low dose.

Newcomer1: Thank you :) feel like I’m a track now and just got to keep heading in the right direction :)^5^

In case of pragmatic discussions, concrete questions are raised by the newcomers and answered by the experienced members. These roles outline a hierarchy, which is a constitutive element of these interactions: the newcomer’s overuse of emoticons can be interpreted as a sign of embarrassment, while the experienced member maintains a quasi-professional façade focusing strictly on substantive issues. The manifest level of the discussion is about the side effects of a medication. However, on the latent level, a similarly important interaction takes place. By reinterpreting the failure of the medication as an issue of dosage, the overall trust in biomedical treatment can be saved. In this case, the experienced member provides a narrative capable of reassuring the newcomer about the rightness of her choice. In this type of discussion, the participants do not step out from the hegemonic biomedical paradigm, instead support is provided for inhabiting its framework by relying on narratives enabling adjustment to its limitations.^6^

The second idealtypical dynamics is exemplified by the following discussion:

Newcomer2: Looking back on my 24 years on this planet I can see a long pattern of fear and avoidance. I have let fear and insecurities rule my life for as long as I can remember. I’m just a coward. Whenever a challenge arises I immediately look for ways to avoid it, I think to myself “How can I get out of doing this scary thing I don’t want to do?” How can I summon the courage to take risk and change my life for the better?

Experienced member 2: I don’t believe you are a coward. Just living in this dangerous world of ours requires unbelievable courage. It is as though most [?] people have become addicted to negative framing and criticism and unable to appreciate any more.

Experienced member 3: Courage is having confidence in yourself and just not caring enough about the consequences. It’s something that can be improved upon. You are not a coward, just pushing yourself outside of your comfort zone takes practice.

Experienced member4: Labeling yourself is hurtful and that particular label deserves to be challenged. You’re not getting a fair trial in your own head, my friend. Let’s try another plausible explanation for your behavior that doesn’t come pre-loaded with a judgment. Maybe your amygdala is hyperactive? Try a little compassion for yourself

Newcomer2: You’re right, I have shamed myself for years lol. Its weird but its so much easier to have compassion for others than for myself. I’ll definitely try to be easier on myself. Thanks for your reply!

This excerpt shows how the depressive self-narrative is reframed according to various interpretative frames. While in case of pragmatic discussion, the boundaries of the initial framing were maintained, in this case the participants challenge the sufferer’s self-interpretation in several ways. Experienced memeber2 provides a contrasting narrative based on a diagnosis of the society: as it is considered to be dangerous and overly critical, what is described as cowardice can be reinterpreted as a logical reaction. From this point of view, the lack of agency introduced by newcomer2 is relativised by highlighting the impossibility of acting in contemporary world. Due to this strategy, a narrative becomes available, which takes away the guilt of the actor by relieving her from the charge of being coward. Experienced member3 relies on a narrative, which also reinterprets cowardice as a shortcoming, which can be improved by proper training. From this perspective, the helplessness is not an essential condition of newcomer2, but a consequence of lacking practice. Experienced member4 highlights a biological narrative. In this case, the aim of reframing is similar to the previous ones; however the substantive argument differs: instead of relativising agency by social or psychological terms, in this case the biological determinedness is evoked. Newcomer2 is grateful for all of these suggestions, independently from their content, which means that the access to alternative perspectives themselves is more important than the substantive explanation. From a phenomenological point of view, it may be argued that these discussions provided opportunity for re-entering a long missing intersubjectivity capable of moving him away from a stuck position. The significance of these engagements is not necessarily the substantive content of the posts, but rather the creation of a multidimensional narrative space enabling the reinterpretation of one’s self-narrative.^7^

The third idealtypical dynamics is exemplified by the following excerpt:

Newcomer3: I always had a problem verbalizing my feelings, so I never did. Actually, this is my first time trying to describe what I’m feeling. I have to reach out to somebody even if that someone is a total stranger on the internet. I’m 25-year-old female and I think I’ve been suffering from depression and anxiety for a very long time. However, I never experienced any big trauma, I grew up with both parents, had a normal childhood, I was never molested or anything like that. That’s why I feel like I’m making this all up, or that my problems are not as serious when compared to other people, so I feel guilty for even thinking I need help.

Elder1: Welcome to the forum. Glad you have posted. You are obviously struggling. It is not too late to seek help. Am just wondering if your uni has a counselling service. If so you can contact them. Please don’t ever think your problems are not as serious as anyone else’s. Also mental illness doesn’t always need an obvious cause. Please keep posting here too.

Elder2: Hey obviously you are having problems and no one’s life is really more or less ‘deserving’ we are all quite messed up humanity that is. And anyway that is the hallmark of depression having low self-esteem. And no you are not a waste of time and please start to talk positive in your life otherwise you become what you fill your head with. just some friendly advice.

Newcomer4: Your story sounds very similar to mine. How do you go about first going to counselling? It is something I have looked at but am terrified of verbalising how I feel. It’s bad enough typing this. I would love to talk through how I feel just online. Does this exist? Would this also help you? Any advice from anyone feeing similar things would be greatly appreciated

Elder3: Hi and welcome. We’re happy to have you in our ranks and as each of us is struggling with some mental health issue, we get how you feel about yourself. Regarding what you wrote about your reaction to seeking professional help, I think you’ve made a step forward by sharing your struggle here. I want to encourage you to continue contributing to this conversation and see if it at all helps to get it out of your head and know others understand.

This discussion highlights a third, latent function of online depression forums besides the reinterpreting of uncertain biomedical explanations and treatments, or the attempts of reframing self-narratives. In this case, newcomer3 and 4 refer to personal experiences inexplicable by common psychological knowledge. This experience is disturbing enough, as the sufferers feel to be lost in the discursive space: they have the impression to be depressed, but cannot justify the self-diagnosis, because they lack the assumed criteria (e.g. trauma). In this situation, the elders apply double strategy: on the manifest level, they try to shepherd the newcomers towards biomedical help, on the latent level they conduct a communication based on the principles of ‘unconditional positive regard’.^8^ This purpose is expressed by the recurring topoi like ‘Glad you have posted’, ‘you are not a waste of time’, ‘and know others understand’. Similar phrases are used in most of the posts of the most experienced members playing the role of ‘recovering helper’. More importantly, they are almost exclusively used by the elders, which indicates their specific functionality.^9^ These speech acts are evoked almost automatically, whenever a new participant attempts to share their experiences in a reluctant way. These ritual elements of interaction are responsible for reinforcing the newcomers that their suffering is recognised and understood not only in an objectifying, but in a hermeneutic way. In this sense, their purpose is to create the initial commitment, which is often missing from new members feeling insecure and unsure about their first steps of initiating intersubjectivity in a virtual platform.

Such communicative pattern can be considered to be ritual due to its repetitive, collective and transformative function. Collective rituals have transformative power due to the experience of ‘collective effervescence’, which enables the restructuring of the self, according to the social frames (Durkheim 1995: 218). Of course, their online version lacks several important components of the original ones, thus having only a contingent transformative power. Nevertheless, online forums still hold the potential of hosting rituals by combining pragmatic, interpretative and approving communicative panels into coherent mechanisms.

### Revealing Latent Dynamics: Ritual Healing in Online Depression Forums

In sum, the topics dominating online forums indicate how the hegemonic biomedical discourse is interpreted by the actors themselves. Furthermore, their dynamics reveal those latent mechanisms, which impact the depressed being in the world itself: besides pragmatic advises, online communities also provide experiences of cognitive restructuring of automatic thoughts and interactions having the potential of re-establishing interaffectivity. In order to explain a latent functionality of these mechanisms, the concept of ‘ritual healing’ is called upon.

From a phenomenological perspective, healing is a transcendental experience, inseparable from the impression of wonder: as illness transforms the whole world into a burdensome horizon, the reconfiguration is always contingent until its occurrence. Thus, it always happens unexpectedly—when one is healed, the element of transcendental intervention is present, as the unsure hope becomes reality (Csordas 2002: 24). As phenomenologically the process of healing is a reconfiguration of the being in the world, it traditionally includes both the influencing of bodily symptoms and the reinterpretation of narratives. In every cultural setting, certain authorities have the right of managing such process, which means that healing relies on power relationships constituting a discursive hegemony capable of effectively influencing the narrative structures (Csordas 2002: 25). While such authority is usually personified by a healer, who is in charge of the process, it is actually the rite itself, which is responsible for the healing. As every power relationship has a subjectificating aspect (Foucault 1982), one’s being in the world can also be changed by relying on a depersonalised power: for this purpose, the subject needs to be subordinated to a discourse capable of reinterpreting the suffering. Accordingly, healing rituals may occur in constellations, where the technologies of power are not connected to concrete persons, but institutions or structures (Prince 1980). The key elements of the rite include three steps: securing mutual predispositions, empowerment of the sufferer and transformation of the being in the world (Csordas 2002: 27).

The latent function of online depression forums can be understood from this description of ritual healing. Even if the religious element and the clearly recognisable healing authorities are missing on structural level, a constellation is constituted, which includes analogies to all three steps identified above. In case of depression, the nature of suffering undermines the horizon of mutual dispositions as depressed subjects lack the ability of entering intersubjective relationships. This challenge is handled by latent forum dynamics securing unconditional positive regard. They are capable of evoking a very limited, but easily accessible interaffectivity, which could serve as a basis of further steps leading out from isolation. The functional equivalent of the second step of ritual healing, the summoning of the transcendental is secured by the pragmatic forum dynamics, which evokes an authority in the form of the scientifically justified biomedical discourse. This is not transcendental at all, but fulfils the function of an unquestionable knowledge. By referring to scientific truths, the universal laws of nature are applied to concrete problems: the depression experienced by the subject appears as the expression of a universal phenomenon. The functional equivalent of transformation is the discursive reframing of the depressed subject’s narratives. In this phase, alternative frameworks are offered to the subject, who has the chance of reflecting on her life from this perspective, to re-evaluate the original attributions of the causes of depression and the potential outcomes as well.

From this perspective, online depression forums can be reinterpreted as spaces holding the potential of hosting secular quasi-healing rituals. In these rituals, both the newcomers, experienced members and elders play a particular role. In order to be able to picture the details of the process, a short case study is introduced summarising the various forum dynamics.^10^

Paul is in his late twenties; he has been dropped out from college, likes to play video games, online streaming and suffers from depression for almost a decade. He has been active in online depression forums since 2013. At the beginning, he only reacted to other’s posts, with the intention of expressing and relating to his own misery: “Yea this happens to me sometimes as well. Some people ask why do you want to be alone, wouldn’t you rather be with people? But what many people don’t understand is that it is entirely possible to be far more lonely with a group of people than you ever could be by yourself”. As he does not get too much feedback, after a few months, he decides to start his own thread about the crisis he went through at that time, the suspension of his studies: “Hello, been lurking on this forum for some months now, and I never started a post because I never felt like what I had to say was important enough to start a whole new topic. I guess until now. So I’m a young college student dealing with some serious depression. Because of this I was thinking of taking the next semester off”. The initial answers to the topic frame the problem as a practical issue, so advices are given about the length and details of suspending the studies, while Paul’s depression is not thematised. Consequently, the topic is ended in a few weeks, as Paul finally decides to leave college.

After this first attempt, Paul experiments with various new threads including his difficulties of interacting with others, practicalities of finding the right therapist, difficulties of friendship and love, medicines. While these topics receive various feedback, it is clear that Paul seeks not simply practical advices, but something more. In those rare moments, when he gets approval and understanding (e.g. feedbacks like: “I can relate” or “I hear you”), he seems to be overjoyed. Gradually he gets to know his way around, which transition is expressed most visibly by the shifting structure of his speech acts: initially he is strictly centred on his own problems, expecting support from the others; later he learns that the key to receive reinforcements is the turning to the others and providing care. The communicative pattern of his last thread clearly exemplifies this transformation.

At this time, he plans to join a training program—the problem is once again helplessness, which is framed this time as ‘cowardice’: “Looking back on my 24 years on this planet I can see a long pattern of fear and avoidance. I have let fear and insecurities rule my life for as long as I can remember. I’m just a coward”. Reinforcing messages start to arrive almost instantaneously: “So sorry that you are feeling stuck! Courage is not the absence of fear. Start with one small step and go from there. It will get easier with practice. Good luck!” or “I think you are a very noble person. Hopefully you will get lots of responses to your post”. At this time Paul has already mastered how to keep the flow of reinforcing messages going, so he reacts to the supportive messages not simply by thanking them, but also returning the compassion, which is exemplified by his reaction to a depressed post claiming that courage can be gained after “everything has been lost”. Paul reacts by reversing the roles and providing comfort himself: “I’m sorry to hear that you’ve lost everything. I can’t imagine what that might mean”.

This role reversal indicates a turning point in Paul’s story as well: while previously he shared mostly concerns and insecurities, at this point his perspective is reconfigured due to the support received. He expresses himself more and more confidentially, opens up to the others, while interiorising the role of the recovery helper: “It’s been about a month since my program started so I guess its time for an update. Waking up in the morning everyday is absolutely the worst part of the day. Everyone in the class is fairly nice and I get along with everyone. The only thing that’s weird is that sometimes I find myself not knowing what to say in conversation. Also, if you’re reading this I hope your depression is improving!”. What needs to be emphasised at this point is the interrelatedness of supporter and supported roles expressed by the extended participation in ritual speech acts. In order to overcome the mood of helplessness, one needs to become a helper. The ritualised communicative patterns of the forums provide an easily accessible way to do just that: as Paul’s example shows, even someone who does not have the competences initially can acquire them by following the conversation and gradually apply its language games. As Paul becomes a ‘recovering helper’ himself, he also opens up for the support provided to him—by repairing the damaged intersubjectivity, the depressed being in the world also transforms.

Of course, rituals require continuous practice to maintain their reinforcing impact: when Paul’s condition improves, he loosens the strings, when his negative thoughts return, he reinforces them. As he states, few months after starting the program: “Hey guys, sorry it’s been so long since I’ve provided an update on my situation. My supervisor and all my coworkers are pretty nice people. But, let’s talk a little bit about my depression. I wake up pretty much everyday in a horrible mood. Aside from the mornings I do find myself thinking about death a lot more. I know in the past few months I have made a lot of changes in my life to help improve myself, but I can’t help but think how pointless everything I’m doing is”. Such relapse—occurring despite the fortunate turn of events in his life—does not find Paul unprepared. At this point, he knows that he could count on the others, all he has to do is joining the ritual communication: “No thank you for continuing to follow my journey and being an active member of this thread. I woudn’t keep posting to this thread if it wasn’t for people like you who provide me with amazing feedback and support”. These closing thoughts express the hardly found security related to the ritual discursive mechanism enabling coping with depression.

Just as living with depression is not a distinct condition, which has a clear start and end, this story also includes various dynamics: Paul introduces his problems; the community establishes a safe and inclusive environment; practical advices are given; successes are celebrated; relapse is experienced; ritual healing is restarted. What needs to be emphasised is that during these discussions the biomedical framing appears only in the background: depression is not discussed as an illness, but rather as an element of being in the world. The ‘elder’ supporters work on this mood by being ready to form intersubjectivities, where Paul may reconfigure his being in the world. As he is reinforced continually about acceptance and understanding, he can share his worries and start reflecting about ways out. Gradually, he also starts to engage in the ritual speech acts, while trying to support those peers, who helped him, but need reinforcement themselves. It is known from the discussion that Paul relies on medical therapy as well, but it remains clear that the online community becomes an essential reference point, which is capable of providing comfort even at the times of relapse.

Cases like this can be interpreted as healing rituals in a sense that they include every key functional components. However, they are not organised into an actual coherent ritual being practised by authorities nominated to the task. Instead they are practised by various independent discursive dynamics. They have the chance of appearing in the same discussion, but this occurrence remains highly contingent, as there are no institutionalised roles being responsible for organising them. The coexistence of the preconditions of ritual healing in online forums is only a structural potential at this moment. However, reflecting on them provides opportunity to improve the chance of this coexistence: by re-establishing intersubjectivity and providing alternative framings, the depressed experience can be reconfigured on a level biomedical intervention is incapable of operating. Overall, depression forums are not only spaces for discussing the consequences of objectification—they also hold the potential of narrative transformations. In this process, the biomedical narrative plays an equally important role as the complementary ones: it may provide the secular substitutive authority needed for ritual healing. Accordingly, late modern illness narratives are not constituted against biomedical ones, but in a hybrid way, by combining and reconfiguring lay and expert knowledge.

This general conclusion concerns not only depression forums, but also highlights a possible new trajectory for contemporary medical anthropology. Besides the critical engagement with biomedicine, those latent structural components have to be reconstructed, which emerge at the fissures of the hegemonic discourses and technologies.^11^ It may be argued that the dysfunctions of biomedicine are not endured passively by the actors, who attempt to complement the interventions with alternative ones. Their efforts may be fragmented, as in case of online depression forums, but certainly have the potential of compensating the missing functional elements. By reflecting on the lay praxes, medical anthropology may just provide the missing link for them to become efficient: it may highlight those structural components, which are already present in a fragmented way, but require further integration.

## Appendix

**Table:**

Thematic cluster	Idealtypical narratives
Ambiguities of biomedicine	Doctors do not share the same experience as patients, so their evaluations needs to be double checked
	Medications are unpredictable, the ideal combination needs to be experimented by patients, even if they prove to be ineffective, pills needs to be taken
	Medication needs to be complemented with other technics of recovery
	Medication can be a trap: side effects and tolerance cost more than the support
	Side effects (e.g. loss of sexual desire, weight gain, tiredness) as seconder causes of depression
	Psychiatry as source of power, being influenced by pharma-company interest, which needs to be resisted or tricked
Bodily causes of depression	Unhealthy nutrition, too much stress, insufficient moving, lack of sleep results in a vulnerable body susceptible to depression
	Inflammation or hidden illness causes depression
Automatic thoughts	Depression as a distinct entity ‘talks’ from the sufferer
	Continuous shame because of inadequacy
	Uncontrollability of the self: emotions and aspects of personality change according to their own logic
	Self-justification of depression: as everything is considered to be bad, only Bad things are perceived, which reinforces the starting point
	Impatience concerning recovery, disappointment caused by the missing of the prematurely expected improvement
Maladaptation, distorted development of personality	Depressed persons learned to punish themselves continuously in order to maintain control through this negative way
	Depression is caused by the inability of dealing with loss or fear
	Depression is caused by a repressed identity (e.g. sexual minority), which cannot be lived freely
	Depression is built around childhood practices of drawing too strict boundaries or disconnect from the world
	Unlearning satisfying activities, forgetting how to feel good
	Gradual alienation from the others, isolation from the loved ones
	The depressed is burdensome, unrelatable company for the others
	Learned fatalism: subjects are casualties of their destiny
	Escapism through addictions or suicide as a last option
	Disappointment in the world incapable of understanding (including formal and informal relationships)
Traumas	Physical violence
	Mental terror
	Overwhelming grief
	Unforgivable guilt
	Betrayal of a close relation
	Discrimination as a member of minority group
Social constraints, expectations	Constant happiness as burdening norm
	The norm of readiness for the others, emotional exploitation
	Overwhelming gender expectations
	Relativising or disdaining the suffering
Personal failures	Experience of superfluity, replicability
	Bad luck of finding friends or partners
	Sense of abnormality, misfit or deviance
	Experience of the ignorance of the others
	Experiences of relative deprivation (material or symbolic)
	Material insecurity (unemployment, threat of homelessness)
Social dysfunctions	Depressive social spaces (school, work, family, partner)
	Exploitative, ignorant social relations (being left alone with overburdening tasks)
	World as a doomed place, depression of climate catastrophe
	Meaninglessness of working and social competition
	Unpredictability of labour market, precariat
	Stigmatisation

## Footnotes

1 While these universalist descriptions of depression reveal the general, existential dimensions of a distorted phenomenological pattern, it should also be emphasised that any particular ‘being in the world’ is also culturally contextualised. The affordances of embodiment, time consciousness, agency and intersubjectivity equally depend on the tools provided by the social actors and institutions. Therefore, a phenomenology of depression implies an ‘ecosocial approach’ (Kirmayer et al. 2017).

2 Besides these ethical measures, also an offer was made about sharing the results with the forum community in a suitable way. Until this day however, no answer has been given by the contacted forum administrators.

3 The observation covered several depression forums, which can be considered to be the largest ones based on the Google search results (for reasons of concealment no links are provided—in case of database-related questions, please contact the author).

4 Similar narrative clusters and asymmetries were identified by a quantitative discourse analysis (Németh et al. 2020).

5 For the protection of anonymity, the citations are slightly modified and no links are provided (in case of database-related questions, please contact the author).

6 Several previous research provides a detailed picture about the implicit reproduction of biomedical discourse in online forums. According to a quantitative study, questions related to medication and therapy constitute a significant and robust segment (approximately 10 %) of the overall discussion (Feldhege et al. 2020). In various ways, these posts are responsible for reiterating and reinterpreting the biomedical discourse.

7 The multidimensionality of mental health recovery narratives is an extensively mapped topic (Llewellyn-Beardsley et al. 2019): the major dimensions include the genre of ‘escape’ (based on the experience of distress and trauma), ‘enlightenment’ (based on the topoi of an epistemic turning point), ‘endeavour’ (based on the narrative of recovery) and ‘endurance’ (based on a survival identity).

8 This term was originally introduced as a specific therapeutic tool (Rogers 1956), but became widely used in psychotherapy. Accordingly, it is among the commonplaces of psy-discourses, and as such, imitated by better informed lay actors—such as recovering helpers—as well.

9 Previous research on online mental health peer support emphasise the potential of challenging stigmas together, while strengthening hope through recovery stories and learning from others’ stories and experiences (Naslund et al. 2016). While these findings are strengthened by our experiences, we argue for complementing them. Peer support does not remain merely information exchange, it also has a ritual element that is the collective reframing of the world, as it is explored in the last section.

10 The case study is based on the overviewing of the online activity of a particular user. As the forums provide opportunity for specifically searching the posts and threads written by any user, long-term discursive dynamics become analysable. In Paul’s case, this included systematically reading and annotating (according to the previously introduced thematic and discursive categories) the first posts belonging to his ‘newcomer’ era and the ones being written by him as an ‘experienced member’. Also, in both cases the related reactions were overviewed in a similar fashion.

11 While others have been advocating the importance of the medical staff getting familiar with lay illness narratives (Heilferty 2009), my argument goes further. Online forums do not have the sole potential of informing medical experts about the first person accounts of the illness—they may be viewed as platforms, where peer support may be facilitated, given that the latent potentials are appropriately revealed.
